# Light-Trapping Engineering for the Enhancements of Broadband and Spectra-Selective Photodetection by Self-Assembled Dielectric Microcavity Arrays

**DOI:** 10.1186/s11671-019-3023-x

**Published:** 2019-05-30

**Authors:** Anni Ying, Lian Liu, Zhongyuan Xu, Chunquan Zhang, Ruihao Chen, Tiangui You, Xin Ou, Dongxue Liang, Wei Chen, Jun Yin, Jing Li, Junyong Kang

**Affiliations:** 1 Collaborative Innovation Center for Optoelectronic Semiconductors and Efficient Devices, Department of Physics/Pen-Tung Sah Institute of Micro-Nano Science and Technology, Xiamen University, Xiamen, 361005 Fujian China; 2 State Key Laboratory of Functional Material for Informatics, Shanghai Institute of Microsystem and Information Technology, Chinese Academy of Sciences, Shanghai, 200000 China; 3 Optoelectronic Division R & D Department, Xiamen Hualian Electronic Corp., Ltd., Xiamen, 361005 Fujian China

**Keywords:** Light trapping, Photodetectors, Self-assembly, Dielectric cavity, Leaky mode

## Abstract

Light manipulation has drawn great attention in photodetectors towards the specific applications with broadband or spectra-selective enhancement in photo-responsivity or conversion efficiency. In this work, a broadband light regulation was realized in photodetectors with the improved spectra-selective photo-responsivity by the optimally fabricated dielectric microcavity arrays (MCAs) on the top of devices. Both experimental and theoretical results reveal that the light absorption enhancement in the cavities is responsible for the improved sensitivity in the detectors, which originated from the light confinement of the whispering-gallery-mode (WGM) resonances and the subsequent photon coupling into active layer through the leaky modes of resonances. In addition, the absorption enhancements in specific wavelength regions were controllably accomplished by manipulating the resonance properties through varying the effective optical length of the cavities. Consequently, a responsivity enhancement up to 25% within the commonly used optical communication and sensing region (800 to 980 nm) was achieved in the MCA-decorated silicon positive-intrinsic-negative (PIN) devices compared with the control ones. This work well demonstrated that the leaky modes of WGM resonant dielectric cavity arrays can effectively improve the light trapping and thus responsivity in broadband or selective spectra for photodetection and will enable future exploration of their applications in other photoelectric conversion devices.

**Electronic supplementary material:**

The online version of this article (10.1186/s11671-019-3023-x) contains supplementary material, which is available to authorized users.

## Introduction

Photodetectors (PDs) are in great demand for enhancing responsivity, which is practically important to its commercial applications, such as optical communication, sensing, and imaging in our daily life. It is well acknowledged that the material extinction in active region of the devices must be high enough to allow the efficient light absorption and photocarrier generation [1]. Hence, the application of advanced light-trapping technology has been considered as the most important approach to realize the efficient photodetection in various broadband PDs [2]. Additionally, the newly raised demands for tunable selective spectral responsivity or multiple band sensing in photodetecting field also need to develop new light-manipulating methods [3–9].

Various optical capture strategies have been developed and employed in optical devices, e.g., the random texture interfaces [10] or three-dimensional (3D) nanostructures [11–14] for sensitivity improvement by fully utilizing the large surface-to-volume ratio and Debye length. Among these 3D light-trapping nanostructures, low Q resonant optical cavity has been considered as the most attractive medium to manipulate light in a broadband range through the multiple resonance modes [15–23]. The main principle is that the whispering-gallery-mode (WGM) resonances in the sphere can enhance the light-matter interactions in the cavity [16, 19, 23] or couple the light into the under-layer substrate through the waveguide mode [17, 20]. Consequently, improved photoelectric conversion efficiency or photo-response can be realized in the corresponding optoelectronic devices [24, 25]. This concept of light trapping in thin-film solar cells by utilizing wavelength-scale resonant dielectric nanospheres was proposed by Grandidier et al. with the aims to enhance the light absorption in the active layer and further photocurrent in the device [15]. Further, significantly enhanced light absorption and power conversion efficiency have been well demonstrated by Cui et al. [16]. The self-assembled dielectric hollow nanospheres, embracing multiple low Q WGM resonances in the visible light region, also have been demonstrated for effective light trapping and short-circuit current density improvement on thin-film solar cells in our previous work [17]. Theoretically, different from the conventionally used optical film technology, this kind of multiple resonances should be possible for the application in PDs towards the specific wavelength manipulation or broadband light-trapping enhancement, but which has not been investigated yet.

In this work, the 3D nanostructured dielectric microcavity arrays (MCAs) were introduced for light-trapping engineering in broadband and specific spectral region on the silicon-based PDs. Here, the wide bandgap semiconductor ZnO was selected as the cavity material, which can be facilely prepared through varieties of physical or chemical methods [26–28]. The hollow spherical ZnO cavity was fabricated using the self-assembled PS nanosphere arrays as template combined with the physical depositing and thermal annealing as reported in our previous work [29]. The significant broadband light trapping was characterized in the optimized ZnO cavities, which was proved to be originated from the WGM resonances by the theoretical calculation. Therefore, a broadband photodetection enhancement was achieved in ZnO MCA-decorated PDs. Meanwhile, because of the multiple WGM resonances, especially the leaky modes in the MCA, the local optical density and the effective absorption at specific wavelength region were promoted in the silicon PDs’ active layer. Consequently, besides the broadband responsivity enhancement, an up-to-25% increment in photo-sensitivity at specific wavelength region (800–940 nm) under the bias of 0 V was successfully achieved. The employment of WGM-enhanced absorption for light management in PDs demonstrated in this work opens the door to various applications in other optoelectronic devices, such as efficient photovoltaics and light-emitting diodes (LEDs).

## Results and Discussion

**Fig. 1:**
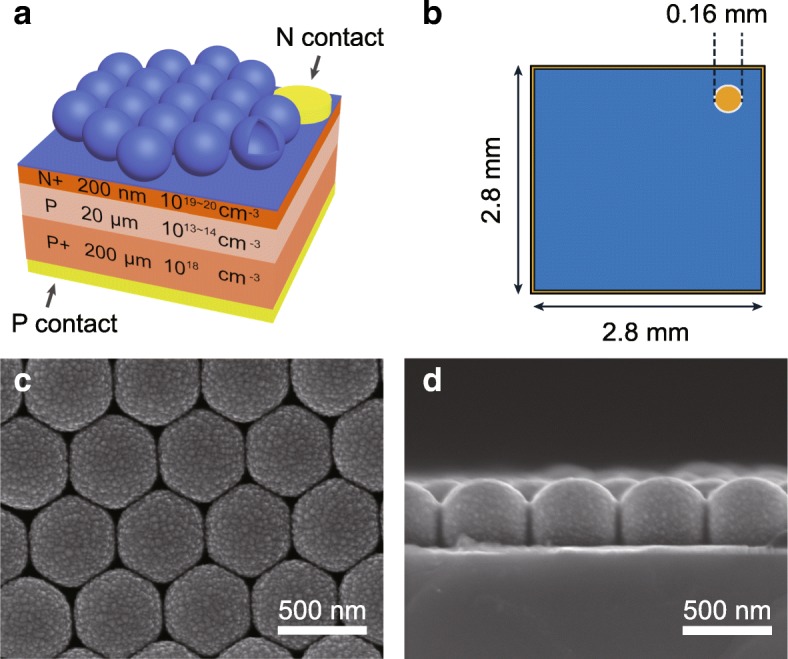
Schematic illustration of **a** the ZnO MCAs decorated PIN PDs and **b** the top view of the PIN device. **c**, **d** The planar and cross-sectional SEM images of the as-fabricated ZnO MCAs on the PIN PD

The cross-sectional and top views of the device structure in the ZnO MCA-decorated PIN silicon PD are schematically shown in Fig. 1a and b, respectively. Here, the as-fabricated ZnO MCAs with the actual core diameter of 470 nm when using the 530-nm-PS nanospheres as template, referring to the experimental details and fabrication processes in (Additional file 1: Figure S1), on the PIN PDs are well ordered in the monolayer arrangement with a hexagonal close-pack as displayed in Fig. 1c. The acceptable spherical shape of the cavities except for the contact area with the substrate can be well recognized in the cross-sectional and titled SEM images of Fig. 1d and Additional file 1: Figure S2a. The smooth inner surface also can be visualized in the internal morphology of this optical cavity as seen in Additional file 1: Figure S2b, which would be understandably beneficial for light resonating in the cavity structure. The actual shell thickness (*T*_shell_) in the cavity was measured to be ~ 40 nm (Additional file 1: Figure S2b). Additionally, clear diffraction color can be seen on the large-scale fabricated ZnO MCA arrays on PIN substrate as shown in Additional file 1: Figure S3a, which originates from the diffraction effect of the ZnO MCA layer that happened at the specific angles satisfying the Bragg’s equation [30]. It is well acknowledged that when cavity parameters (e.g., diameter and thickness) match with the light wavelength, the whispering-gallery-mode (WGM) resonances would be generated. Therefore, in this kind of MCA-decorated PIN PDs, the light confinement and coupling into the active layer of PD through the leaky modes [30] and the consequent light-trapping enhancement in the devices can be expected.

**Fig. 2:**
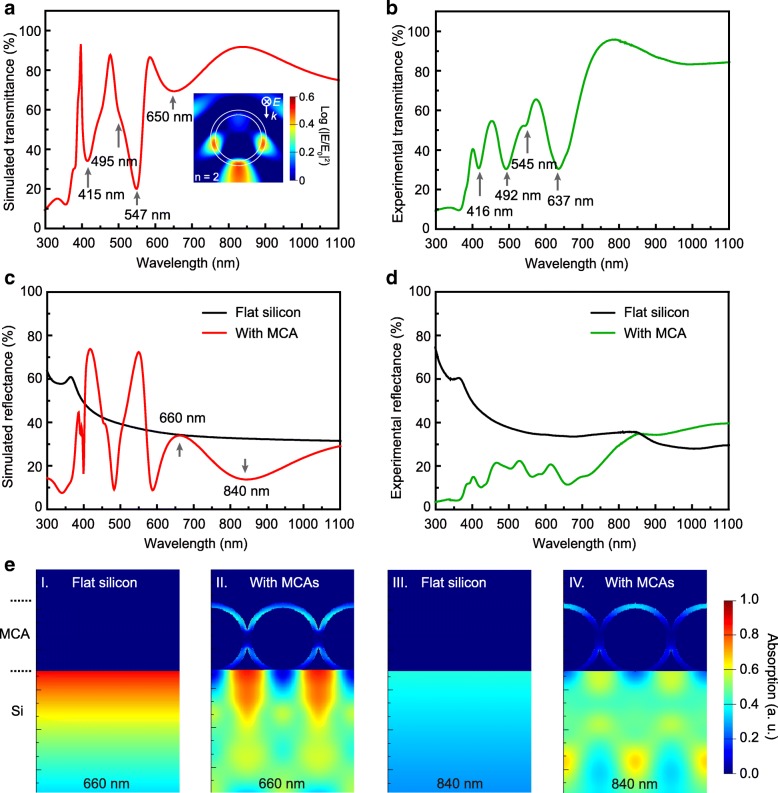
**a** Theoretical and **b** experimental transmission spectra of the MCAs on the sapphire substrate. **c**, **d** Theoretical and experimental reflection spectra of the MCAs on silicon substrates comparing with those on the bare silicon. **e** The absorption profile in the silicon substrate with and without MCA decoration under the on-resonance (660 nm) and off-resonance (840 nm) light excitations

In order to verify the light confinement and trapping properties of the fabricated ZnO MCAs, FDTD simulated transmission spectrum for the ZnO MCAs on the sapphire substrate as a simplified case was firstly examined and compared with the experimental results, as shown in Fig. 2a and b. Several distinguished valleys can be well resolved at wavelengths of 415, 495, 547, and 650 nm in the simulated transmission spectrum. Because of the intrinsic band-edge absorption of ZnO, no resonance appeared in the UV region where the wavelength is shorter than 380 nm. Undoubtedly, these valleys in transmission spectrum originate from the series of supported WGM resonances in the ZnO MCAs and can be well identified by their corresponding near-field distribution patterns under each resonance peak, as shown in Additional file 1: Figure S4. The typical resonance pattern for the second order of WGM resonance near 650 nm was selectively shown in the inset of Fig. 2a. An intensified field distribution was clearly resolved around the cavity, which is known as the leaky mode [31] and would be subsequently favorable to the light radiating into the underlying active layer of the devices. The experimental transmission spectrum agrees well with the simulated one at the corresponding resonance wavelengths except for a little shift of wavelength peaks at 416, 492, 545, and 637 nm, as shown in Fig. 2b. These WGM resonances in the MCAs produced a broad angle scattering [32] of the incident light, exhibiting as a valley in the transmission spectra near the resonance wavelength.

This scattering effect on ZnO MCAs decorated Si substrate also can be well evidenced by the simulated reflection spectrum as shown in Fig. 2c, where series of peaks can be found which matched well with the resonance valleys shown in the transmission spectra [33]. Additionally, it was found that a broadband anti-reflection effect was successfully achieved on the MCA-decorated silicon substrate when compared with the bare silicon. The experimental reflection spectrum on ZnO MCA-decorated silicon substrate (Fig. 2d) also shows the similar anti-reflection effect and resonance peaks to the theoretical results, except for a much lower resonance quality (Q) which might be caused by the non-ideal spherical structure and the existed defects within the experimentally prepared MCAs. However, this decreased resonance quality might be further conducive to the anti-reflection in the short wavelength region (< 550 nm), which would be much beneficial for the broadband light trapping on the corresponding devices as evidenced in the previous work [16, 34].

With comparing to the reflection from the bare silicon surface, both the theoretical and experimental reflection spectra from the MCA-decorated silicon well demonstrated that the supported series of WGM resonances can be used for light trapping by utilizing the leaky modes. However, interestingly, it was noteworthy that the mostly decreased reflection happened in the off-resonance region rather than the on-resonance peaks. Further simulation well indicated that the strong absorption enhancement can be successfully realized in the MCA-coated silicon substrate under the off-resonance band (840 nm) compared with that on the bare silicon, while much lower absorption profile was obtained under the on-resonance illumination (660 nm), as shown in Fig. 2e (the detailed simulation set up was shown in Additional file 1: Figure S5). This result infers that the WGM resonance, especially the resonance with high-quality factor in some special wavelength positions, might also scatter the light back [35], which is unfavorable for the light-trapping enhancement. The extracted near-filed distribution shown in Additional file 1: Figure S6 also evidenced that a large amount of optical power was scattered back due to the resonance, leading to a decreased absorption profile in the active layer while comparing with bare silicon under the on-resonance wavelength illumination.

**Fig. 3:**
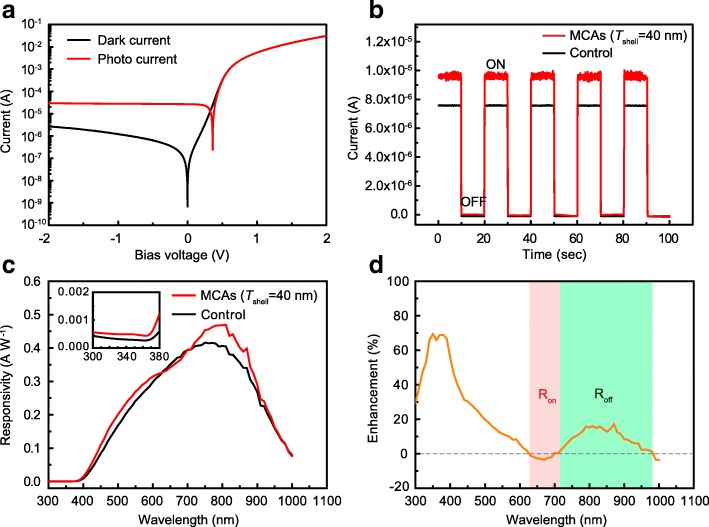
**a** Current-voltage (IV) curves for the fabricated silicon PIN PDs under dark and light illumination (850 nm LED, 1.2 mW cm^−2^). **b** Comparison of the current response under 850 nm LED light illumination and **c** the wavelength-dependent photo responsivity in the devices with and without (control) MCAs’ decoration. The partial enlargement in shorter wavelength region (< 380 nm) was shown in the inset. **d** The corresponding enhancement ratio calculated from **c**, in which the on-resonance (*R*_on_) and off-resonance (*R*_off_) region referred to the reflection spectra marked as light red and light green in the background, respectively

The functionality of the light-trapping MCA layer on silicon PIN PDs was then evaluated by characterizing the photo-response of the devices. As shown in the typical I–V response of Fig. 3a, a satisfying photodiode characteristic was verified in the as-fabricated silicon PIN PD devices under both the dark condition and light illumination. Significantly, with the decoration of MCAs, an enhanced photo-response by up to ~ 25% can be realized on the PDs comparing with that in the only silicon PIN PDs under 850 nm light illumination (as seen in Fig. 3b). The wavelength-dependent photo-responsivity as shown in Fig. 3c presents a dramatically enhanced photo-response within a broadband spectrum nearly over the whole visible and near-infrared (IR) region after decorating the MCAs on the devices. The enhancement ratio was calculated and is shown in Fig. 3d. It can be seen that only within the wavelength region from 625 to 695 nm with the center valley located at ~ 660 nm there is no enhancement, which just matched well with the second-order (*n* = 2) WGM resonance (peak wavelength at ~ 640 nm) as seen in the transmission spectra (on-resonance region) of Fig. 2b. While within the mostly used near-infrared (IR) region (~ 800 to ~ 980 nm) for silicon PDs, obviously enhanced responsivity by up to ~ 17% was successfully accomplished. Coincidentally, this wavelength region also lay at the off-resonance region as mentioned above. The results were well consistent with the simulation results where absorption enhancement could not be enhanced under the on-resonance illumination while obviously enhanced absorption can happen in the off-resonance region, as shown in Fig. 2e. However, for the short wavelength region (< 600 nm), the significant enhancement in absorption, as well as the photo-response, still can be obtained, which matched well with the remarkable anti-reflect properties for the MCAs on silicon presented in Fig. 2d. As discussed above, the actual much low resonance quality in cavities within this region should be the main reason for the broadband light trapping which is independent of the on-resonance or off-resonance.

**Fig. 4:**
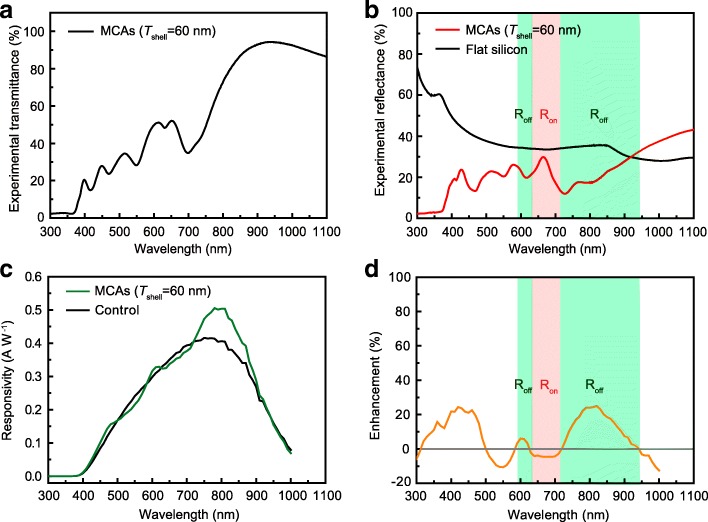
**a** Experimental transmission spectrum of the MCAs on sapphire substrate with the shell thickness of 60 nm. **b** The corresponding reflection spectra of MCAs on a silicon substrate, compared with the bare silicon substrate. **c** The photo-responsivities in the device with or without (control) MCAs’ decoration under 850 nm LED light illumination. **d** The corresponding enhancement ratio calculated from **c**. The background within the on-resonance and off-resonance region in **b** and **d** referring to the reflection spectra in **b** was highlighted in light red and light green, respectively

The above results well demonstrated that the light-trapping properties via the WGM microcavity are highly related to the resonance quality, which is dependent on the cavities’ parameters. In order to further verify the enhancement mechanism mentioned above and manipulate the responsivity enhancement on devices in specific wavelength region, such as the widely used near-infrared (IR) region detecting for communication or sensing, the WGM resonances in MCAs were regulated by controlling the cavities’ size. For the shell structure cavity adopted in this work, the effective optical length can be easily increased by thickening the shell layer [36]. As shown in Fig. 4a, by increasing the shell thickness to 60 nm, much more resonance modes were observed in the transmission spectrum of the MCAs. These resonance modes also can be assigned to the corresponding WGM resonances by means of the theoretical simulation, as shown in Additional file 1: Figure S7. Comparing with the MCAs in shell thickness of 40 nm (Fig. 2b), the same resonance mode exhibits an understandable redshift due to the increased effective cavity length. The experimental reflection spectra in Fig. 4b also matched well with the transmission spectrum. Different from the experimental reflection spectra for the MCAs with a shell thickness of 40 nm shown in Fig. 2d, the actual resonance is more distinguishable indicating the higher resonance quality, which means that the backscattering effect might be stronger and not in favor of the light trapping. The wavelength-dependent responsivity curves are shown in Fig. 4d well demonstrate this inference, where responsivity in specific wavelength regions has been enhanced while some other regions were decreased. From Fig. 4d, it can be noted that the mostly enhanced region consistently happened in the off-resonance area while decrementing region located in the on-resonance area. Additionally, compared to the MCAs decorated PDs with shell thickness of 40 nm (shown in Fig. 3d), much higher responsivity enhancement was achieved within the region of 800–980 nm, which is mostly used in communication and sensing for silicon PDs. An up to ~ 25% enhancement can be achieved at the wavelength of 820 nm, as shown in Fig. 4d. This much stronger enhancement should have originated from the higher resonance quality for the second-order WGM of the MCAs, leading to the higher light-trapping effect through the leaky mode of WGM resonance in this wavelength region. The much lower reflectance intensity in this wavelength region well explained this significant enhancement in light trapping, as well as the responsivity, as shown in Fig. 4b when comparing with the reflection spectrum in Fig. 2d for the MCAs with a shell thickness of 40 nm. Additionally, this enhancement also mostly happened at the off-resonance region.

While for the on-resonance region from ~ 640 to 710 nm as shown in Fig. 4d (background was marked as light red), obviously decreased responsivity was obtained reasonably due to the backscattering effect induced by the high resonance quality for this resonance mode, as discussed above. Similar as the MCAs with a shell thickness of 40 nm, strong enhancement still can be realized in the short wavelength region (< 500 nm) most likely because of the much lower resonance quality and higher anti-reflection effect. The stability performance for these enhancements by the light-trapping engineering also has been further evaluated by examining the photo-response for the same device storing in ambient air for 1 year, which show nearly no decay in the current response compared with the control one under the same test conditions, as seen in Additional file 1: Figure S8.

## Conclusions

In conclusion, a new strategy was proposed for light absorption improvement within broadband and specific wavelength region for photodetectors (PDs) by utilizing the multiple WGM resonances generated in ZnO microcavity arrays (MCAs). With the decoration of the facilely prepared dielectric microcavity arrays (MCAs) on the silicon-based PIN PDs, a broadband light trapping and photo-responsivity enhancement were successfully achieved covering nearly the whole ultraviolet-visible near-infrared (300–1000 nm) region. Theoretical and experimental results indicated that the leaky mode radiation of the WGM resonances, which most effectively work in the off-resonance region, is the main enhancement mechanism for light trapping. With further manipulating the WGM resonance peaks and resonance quality by increasing the shell thickness of cavities, specific light trapping and responsivity enhancement were achieved in the mostly used communication and sensing region (800–980 nm) with the maximum improvement of up to ~ 25% at 820 nm. This work well demonstrated a low-cost and good compatibility method to improve the light trapping and thus responsivity with broadband or selective spectra for photodetection by introducing the leaky mode of WGM resonant dielectric cavity arrays. The light manipulation approach employed in this work provides an important guide for designing micro- and nanomaterial architectures to facilitate the novel applications within a specific wavelength range in optoelectronic devices.

## Methods/Experimental

### Fabrication Process of PIN PD Devices

The PIN PDs were fabricated on a 200-μm-thick p-type (100) silicon substrate purchased from WaferHome [37] with the resistivity of 0.001 Ω cm. A 20-μm-thick intrinsic layer was epitaxially grown on the substrate. Then, n-type phosphorus-ion implantation with an implantation dose of 1 × 10^16^ cm^−2^ and an energy of 160 keV was performed on the intrinsic layer to form the final PIN device structure. Before the decoration of the MCA structures, the PIN wafer was standardly cleaned to remove the surface residual organic matters and metal ions. Finally, the chip-fabrication processes were carried out with the designed photosensitive region of 2.8 mm × 2.8 mm. A 100-nm-thick aluminum electrode in a diameter of 160 μm on the n-type surface and a 50-nm-thick Au film with 5-nm Ti bonding layer on the back side were sputtering deposited (Explorer-14, Denton Vacuum) to form a metal ohmic contact.

### Fabrication Process of ZnO MCA Layer

The ZnO MCAs were produced using the polystyrene (PS) nanospheres as the template followed by sputtering deposition of ZnO film, and the PS nanospheres were finally removed by thermal annealing [29]. Commercial PS nanospheres purchased from Nanomicro (Suzhou Nanomicro Technology Co., Ltd.) in the diameter of 530 nm were used as the template material to fabricate ZnO microcavity arrays. The shell of ZnO thin films in different thicknesses (~ 40 and ~ 60 nm) was controlled by adjusting the different deposition durations.

### Characterizations

The morphology and structure were characterized by Hitachi S-4800 field emission scanning electron microscope (FE-SEM). Experimental transmission and reflection spectra data were collected by Varian Cary 5000 UV-Vis-NIR spectrophotometer. The photocurrent and IV characteristics of the devices were measured on an electrochemical workstation (CHI660D) equipped with a room-temperature probe station and LED light sources. The external quantum efficiency (EQE) of the devices under 0 bias were measured using an optical power meter (Newport, 2936-R), which equipped with a light source (Newport, 66,920) and a monochromator (Cornerstone 260, Newport). Simulated transmission/reflection spectra and near-field distribution were extracted by a FDTD simulation package (FDTD Solutions, Lumerical Inc.).

## Additional File

Additional file 1: **Figure S1.** Fabrication of ZnO MCAs on Si PIN substrate. **Figure S2.** Detailed morphology of ZnO MCA arrays on PIN substrate. **Figure S3.** Large-scale ZnO MCA arrays on PIN silicon substrate. **Figure S4.** Near-field distribution patterns of ZnO MCA with shell thickness of 40 nm. **Figure S5.** Simulation method and setup for the absorption profile. **Figure S6.** Comparison of the absorption profile and near-field distribution for the MCAs on silicon substrates under on/off-resonance wavelengths. **Figure S7.** Near-field distribution patterns of ZnO MCA with shell thickness of 60 nm. **Figure S8.** Response stability of MCA-decorated PIN PD. (DOCX 3687 kb)

## Data Availability

All data generated or analyzed during this study are included in this published article and its supplementary information files.

## Abbreviations

3D: Three dimensional
EQE: External quantum efficiency
IR: Infrared
IV: Current-voltage
MCAs: Microcavity arrays
PDs: Photodetectors
PIN: Positive-intrinsic-negative
PS: Polystyrene
*R*_off_: Off-resonance
*R*_on_: On-resonance
*T*_shell_: Shell thickness
WGM: Whispering-gallery-mode
