# Supplementary material for: Light-Trapping Engineering for the Enhancements of Broadband and Spectra-Selective Photodetection by Self-Assembled Dielectric Microcavity Arrays
Source: Nanoscale Res Lett. 2019 May 30;14:187. doi: 10.1186/s11671-019-3023-x (PMC6542964; doi:10.1186/s11671-019-3023-x)
Supplement: Supplementary file 1 — Figure S1. Fabrication of ZnO MCAs on Si PIN substrate. Figure S2. Detailed morphology of ZnO MCA arrays on PIN substrate. Figure S3. Large-scale ZnO MCA arrays on PIN silicon substrate. Figure S4. Near-field distribution patterns of ZnO MCA with shell thickness of 40 nm. Figure S5. Simulation method and setup for the absorption profile. Figure S6. Comparison of the absorption profile and near-field distribution for the MCAs on silicon substrates under on/off-resonance wavelengths. Figure S7. Near-field distribution patterns of ZnO MCA with shell thickness of 60 nm. Figure S8. Response stability of MCA-decorated PIN PD. (DOCX 3687 kb) [file 11671_2019_3023_MOESM1_ESM.docx]

**Additional file 1**

**Light trapping engineering for the enhancements of broadband and spectra-selective photodetection by self-assembled dielectric microcavity arrays**

Anni Ying,^a^ Lian Liu,^a^ Zhongyuan Xu,^a^ Chunquan Zhang,^a^ Ruihao Chen,^a^ Tiangui You,^b^ Xin Ou,^b^ Dongxue Liang,^a^ Wei Chen,^c^ Jun Yin,*^a^ Jing Li*^a^ and Junyong Kang ^a^

^a^ Collaborative Innovation Center for Optoelectronic Semiconductors and Efficient Devices, Department of Physics /Pen-Tung Sah Institute of Micro-Nano Science and Technology, Xiamen University, Xiamen, Fujian 361005, China;

^b^ State Key Laboratory of Functional Material for Informatics, Shanghai Institute of Microsystem and Information Technology, Chinese Academy of Sciences, Shanghai 200000, China;

^c^ Optoelectronic Division R&D Department, Xiamen Hualian Electronics Corp.,Ltd, Xiamen, Fujian 361005, China.

*Email: jyin@xmu.edu.cn, lijing@xmu.edu.cn

**S1. Fabrication of ZnO MCAs on Si PIN substrate.**

**
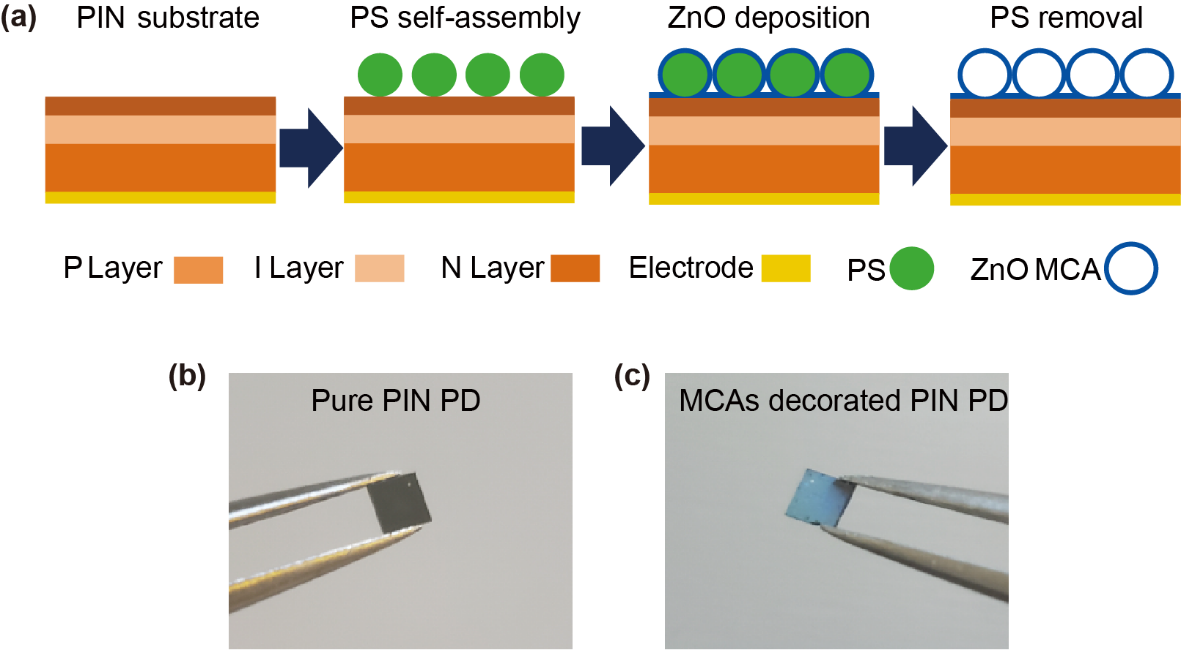
**

Additional file 1: Figure S1. (a) Schematic diagram of the fabrication processes of ZnO MCAs on Si PIN substrate. The physical picture of (b) the as-fabricated silicon PIN PD device and (c) ZnO MCAs decorated PIN PD.

**S2. Detailed morphology of ZnO MCA arrays on PIN substrate**


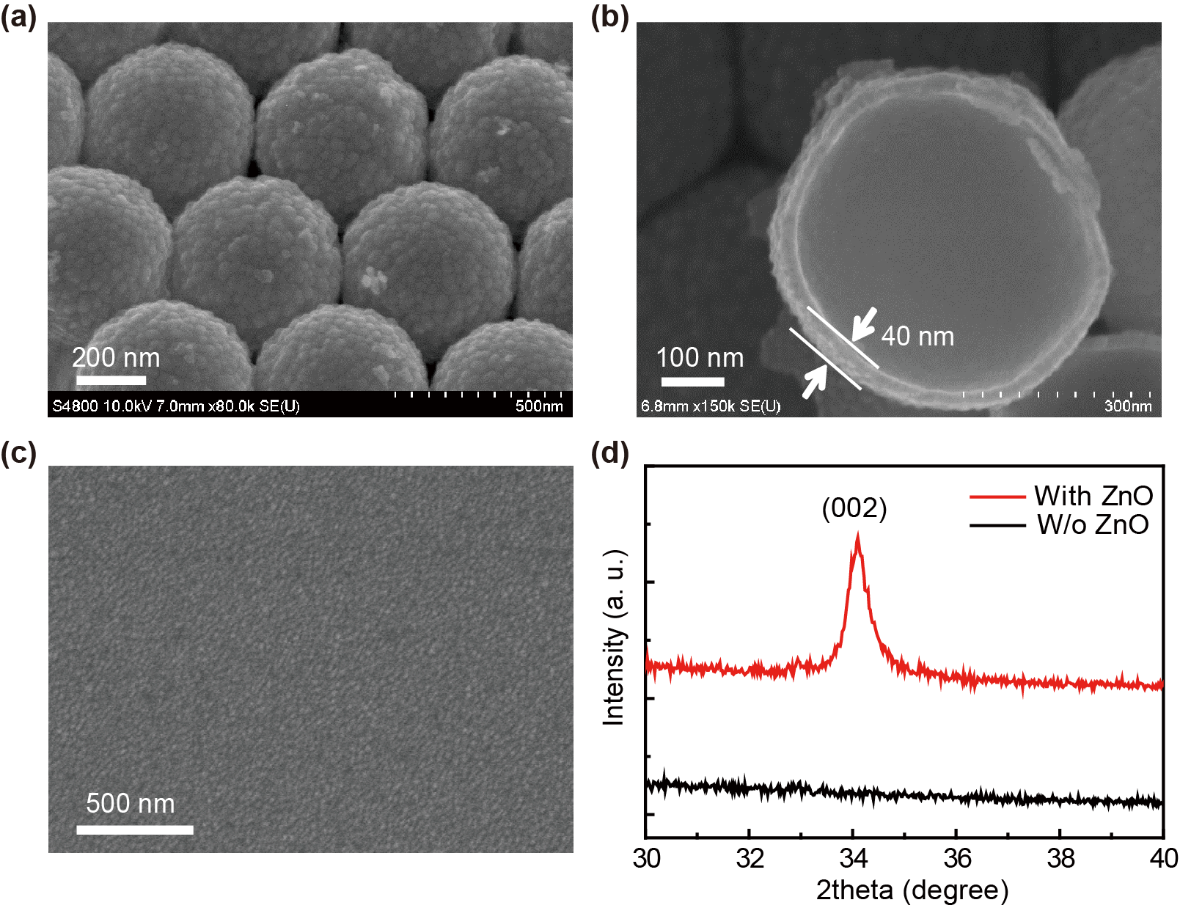


Additional file 1: Figure S2. (a) SEM images of the as-fabricated ZnO MCAs in high magnifications. The colloidal sphere template was assembled using the 530 nm PS nanospheres and without other treatment. The actual core diameter of the fabricated ZnO micro cavity was measured to be about ~470 nm and the shell thickness was measured to be ~40 nm as shown in (b). (c) SEM image of the sputtered ZnO film on silicon substrate and (b) corresponding XRD pattern compared to the bare silicon substrate. It can be seen that the (002) plane diffraction dominated the crystal structure, indicating a satisfied crystallinity for the ZnO films.

**S3. Large scale ZnO MCA arrays on PIN silicon substrate**

**
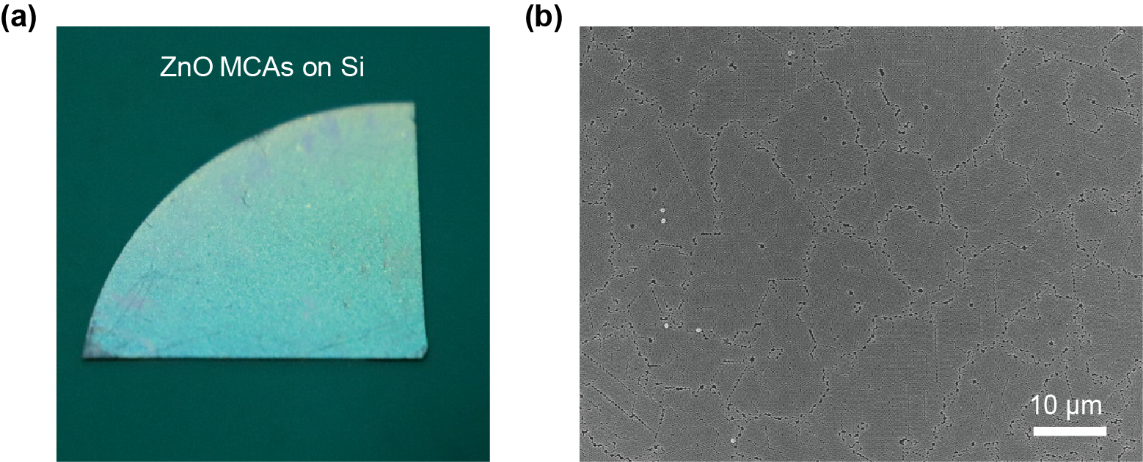
**

Additional file 1: Figure S3. (a) The photograph of the fabricated monolayer ZnO MCAs on the PIN substrate. Apparent diffraction color can be seen on the fabricated ZnO MCAs in (a), which should come from the elastic coherent scattering from the ZnO MCAs and happens at the specific angles that satisfy Bragg's equation. (b) The SEM images of the as-fabricated ZnO MCAs in low Magnifications.

**S4. Near-field distribution patterns of ZnO MCA with shell thickness of 40 nm**


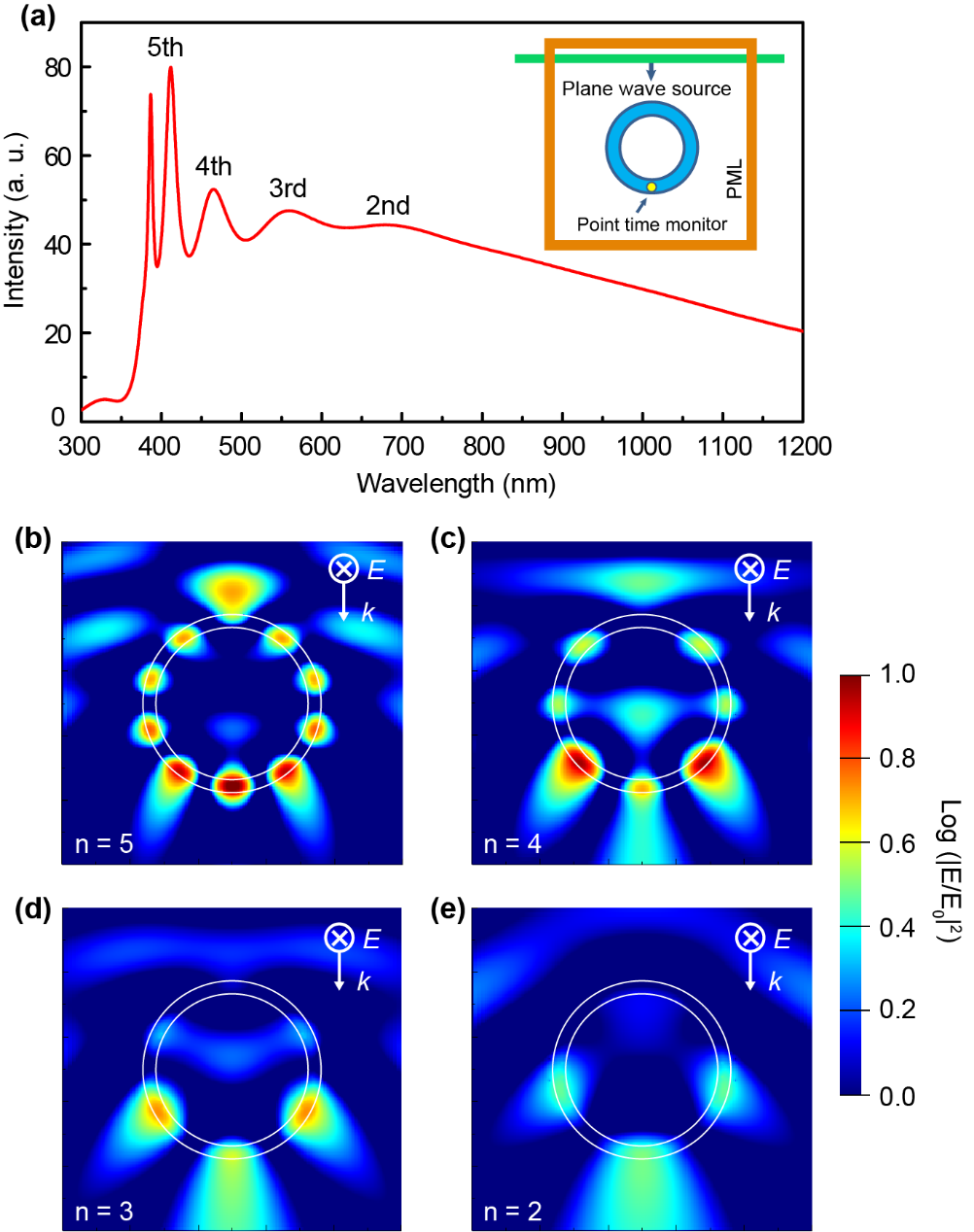


Additional file 1: Figure S4. (a) Simulated resonance spectra for a single ZnO microcavity with core diameter of 470 nm and shell thickness of 40 nm, which are consistent with the actual values of the fabricated cavities. The simulation setup was schematically shown in inset. Clear resonance modes can be resolved in the spectra, and the corresponding peaks were located at 412, 465, 555, and 658 nm, which matched well with the resonance valleys in the simulated transmission spectrum (peaks located at 415, 495, 547 and 650 nm). (b)-(e) show the corresponding extracted near-field distribution for the single ZnO microcavity at the incident wavelengths of 412, 465, 555 and 658 nm, separately. According to the near-field patterns, the resonances can be assigned to the 5th, 4th, 3rd, and 2nd TE mode, respectively. The direction and polarization of incident light are shown in the patterns.

**S5. Simulation method and setup for the absorption profile**

**
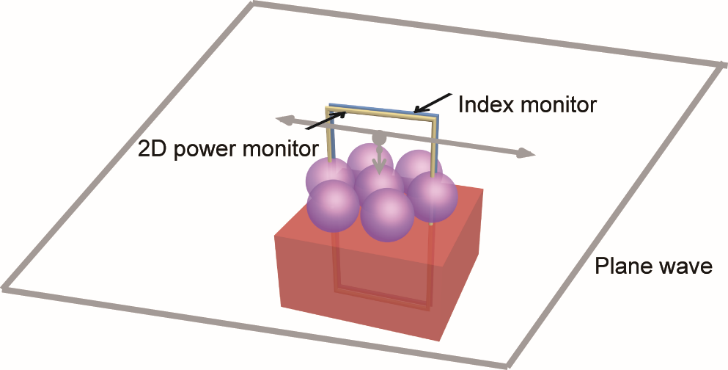
**

Additional file 1: Figure S5. Schematic illustration of the simulation setup for the absorption profile. Here, the power absorption per unit volume (*P*_abs_) can be calculated from the formula below:


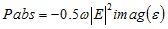


where *ε* represents the permittivity, *E* the electric field intensity and *ω* the angular frequency of incident light. Above quantities all can be extracted in the FDTD simulation. The simulation model is composed of an Air-ZnO MCAs-Si stacks and plane wave source was used as the incident light. A 2D power monitor was applied to measure the electric field intensity and an index monitor was used to measure the permittivity. Both monitors were set as the same size. After running the simulation with FDTD and the analysis script, a number of matrixes containing the electric field intensity *E*, real part of the refractive index and imaginary part of the refractive index can be subsequently obtained. Thus, the absorption profile can be calculated and plotted as shown in Figure 2e.

**S6. Comparison of the absorption profile and near-field distribution for the MCAs on silicon substrates under on/off-resonance wavelengths**


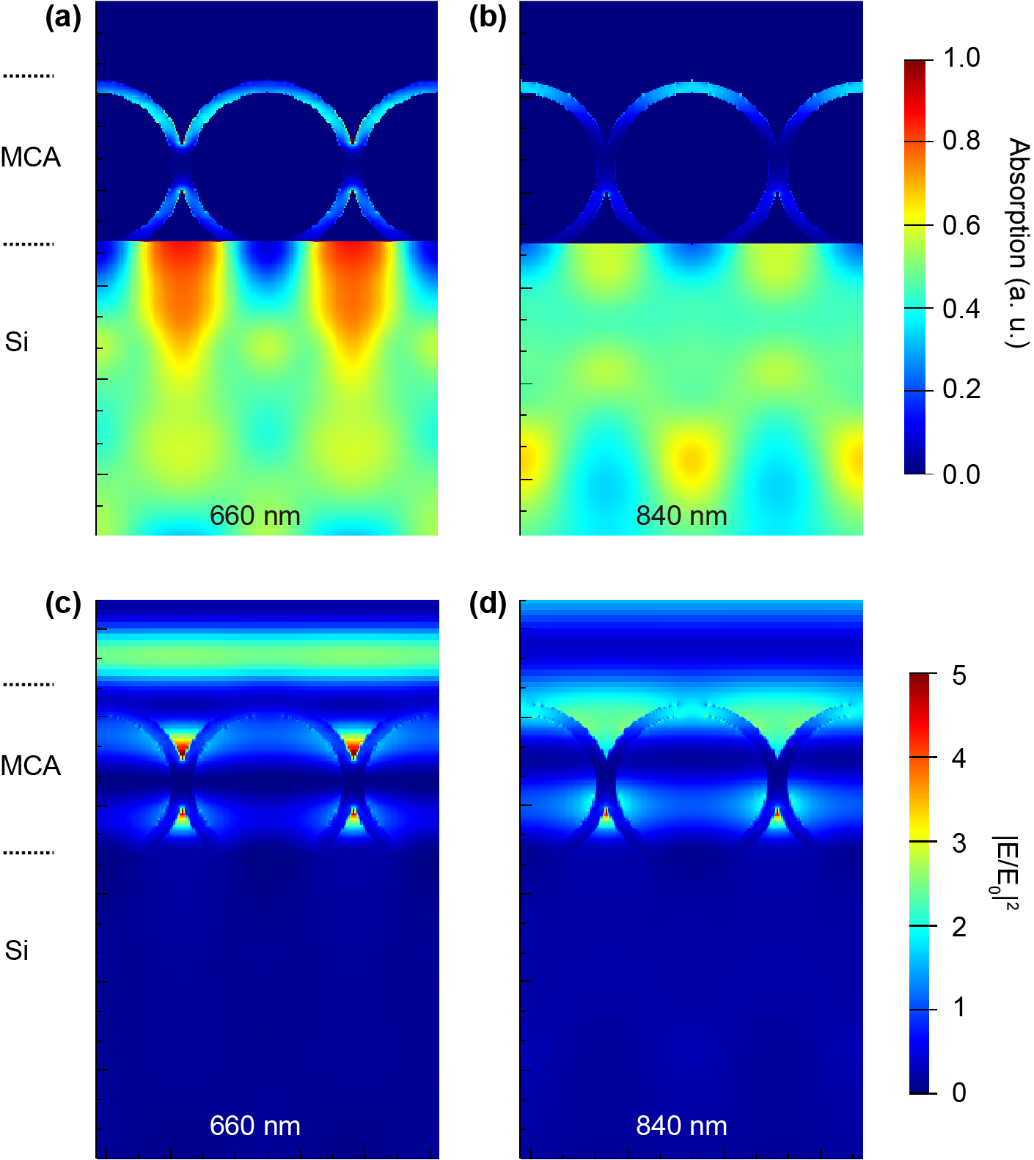


Additional file 1: Figure S6. (a) and (b) show the absorption profiles for the MCAs on silicon substrates under on/off-resonance peaks (660 nm and 840 nm); (c) and (d) show the corresponding near-field distribution under the on-resonance illumination (660 nm) and off-resonance illumination (840 nm), respectively. Comparing with the off-resonance illumination, a strong back scattering induced intensified near-field distribution can be clearly seen on the MCAs under the on-resonance excitation, which well explained the decreased absorption profile in the active layer comparing with that in bare silicon and under the on-resonance wavelength illumination, as shown in Figure 2e.

**S7. Near-field distribution patterns of ZnO MCA with shell thickness of 60 nm**

**
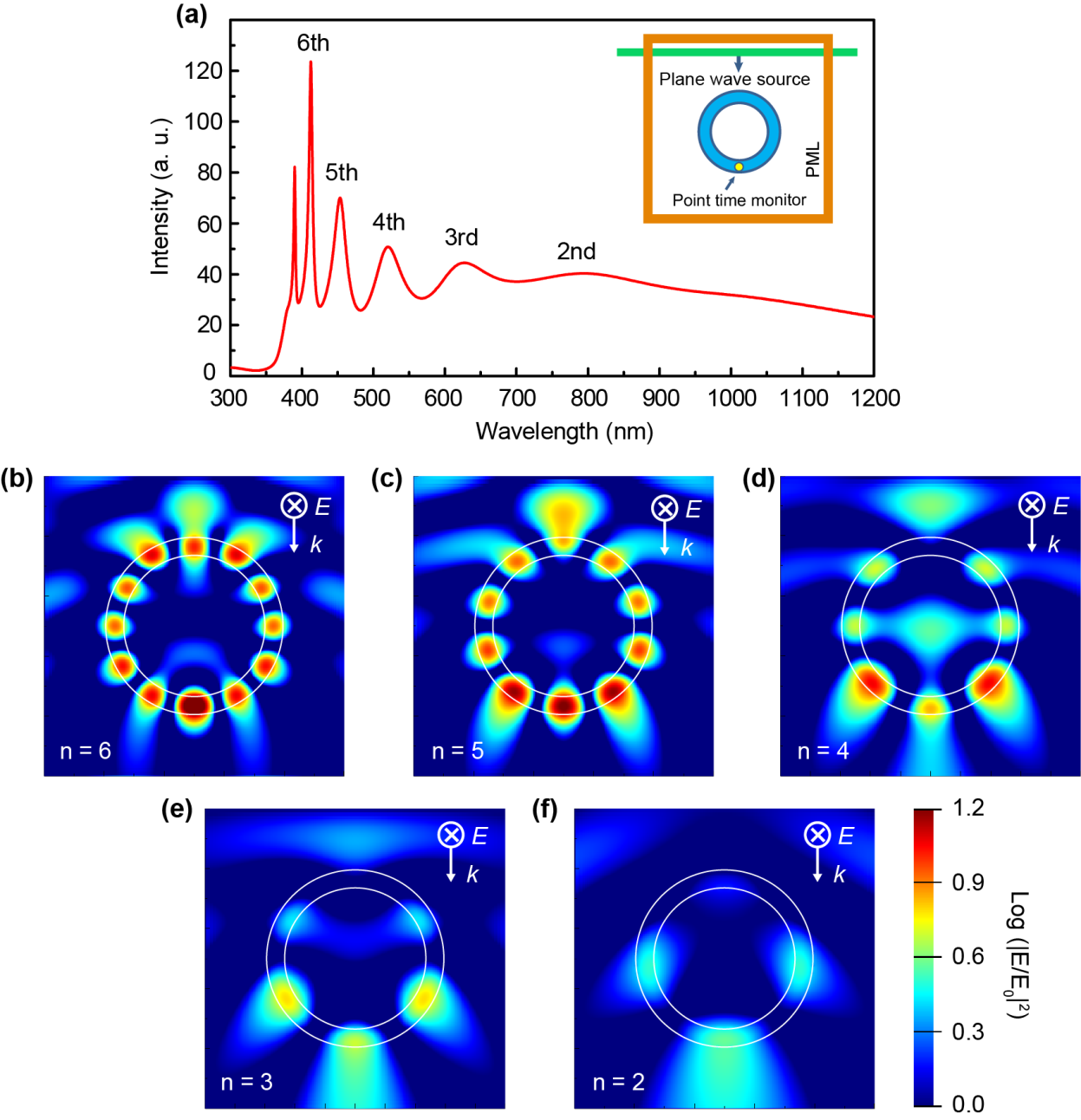
**

Additional file 1: Figure S7. (a) Simulated resonance spectra for a single ZnO microcavity with core diameter of 470 nm and shell thickness of 60 nm, which was consistent with the actual values. Clear resonance modes can be resolved in the spectra, and the corresponding peaks were located at 412, 454, 521, 628 and 780 nm, which matched well with the resonance valleys in the simulated transmission spectra (peaks located at 416, 469, 548, 632 and 720 nm). (b)-(f) show the corresponding extracted near-field distribution for the single ZnO microcavity at the incident wavelengths of 412, 454, 521, 628 and 780 nm, respectively. According to the near-field patterns, the resonances were assigned to the 6th, 5th, 4th, 3rd, and 2nd TE optical resonance mode, respectively. By increasing the shell thickness to 60 nm, much more resonance modes were observed in transmission spectra for the MCAs comparing with that with shell thickness of 40 nm.

**S8. Response stability of the MCAs decorated PIN PDs**


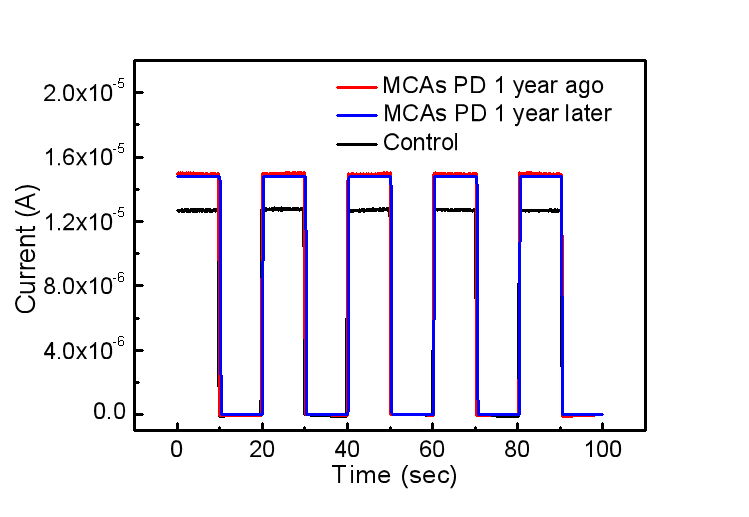


Additional file 1: Figure S8. Comparison of the current response for the MCAs decorated device before and after 1 year under 850 nm LED light illumination, compared with the control device.
